# Mislocalisation of FLT3-ITD receptor contributes to MV4-11 leukaemia cell resistance to antibody-drug conjugate

**DOI:** 10.1080/14756366.2026.2638027

**Published:** 2026-03-05

**Authors:** Wariya Nirachonkul, Mark P. Farrell, Thomas J. Tolbert, Siriporn Okonogi, Singkome Tima, Songyot Anuchapreeda, Sawitree Chiampanichayakul, Teruna J. Siahaan

**Affiliations:** a Department of Medical Technology, Chiang Mai University, Chiang Mai, Thailand; b Department of Medicinal Chemistry, The University of Kansas, Lawrence, KS, USA; c Department of Pharmaceutical Chemistry, The University of Kansas, Lawrence, KS, USA; d Department of Pharmaceutical Sciences, Chiang Mai University, Chiang Mai, Thailand; e Center of Excellence in Pharmaceutical Nanotechnology, Chiang Mai University, Chiang Mai, Thailand; f Cancer Research Unit of Associated Medical Sciences (AMS CRU), Chiang Mai University, Thailand

**Keywords:** FLT3-internal tandem duplication (FLT3-ITD), antibody-drug conjugates (ADCs), acute myeloblastic leukaemia (AML), MV4-11, FLT3-MMAE conjugate

## Abstract

FMS-like tyrosine kinase 3 (FLT3/CD135) regulates haematopoiesis and is frequently mutated as FLT3-internal tandem duplication (FLT3-ITD) in acute myeloid leukaemia (AML), associated with poor prognosis. Although FLT3 inhibitors show clinical benefits, resistance remains a challenge. This study hypothesises that antibody-drug conjugate (ADC) efficacy depends on distinct FLT3 trafficking mechanisms in FLT3-wt and FLT3-ITD cells. Confocal imaging showed that in THP-1 (FLT3-wt) cells, FLT3 mAb trafficked to lysosomes, while in MV4-11 (FLT3-ITD) cells, it accumulated in the Golgi. To evaluate the impact of this trafficking difference, we synthesised an anti-FLT3 mAb-MMAE, linked via a Val-Cit-PAB linker at the Fc N-glycan, which exhibited lower cytotoxicity in MV4-11 than THP-1 cells, indicating that the impaired lysosomal trafficking of FLT3-ITD limits drug release and reduces ADC potency. These findings highlight that effective lysosomal targeting is essential for ADC activity and suggest that optimising linker design or restoring lysosome trafficking may enhance FLT3-targeted ADC in AML.

## Introduction

Targeted drug delivery strategies have been successfully applied to reduce the side effects of anticancer agents by directing cytotoxic drugs specifically to tumour cells while avoiding normal cells. This selective delivery minimises off-target effects and improves patient tolerance compared to conventional chemotherapy. Among various targeted therapies, antibody-drug conjugates (ADCs) have emerged as one of the most successful therapeutic agents due to their high specificity and low side effects. ADCs are composed of a monoclonal antibody (mAb) linked to a potent cytotoxic payload through a chemical linker. The mAb selectively recognises a target protein or receptor on the cancer cell surface with no or low non-specific binding to the healthy cells. This binding is followed by the uptake of ADCs into the cell through endocytic pathways via the endosomal-lysosomal pathway. Within the lysosome, the ADCs are degraded to release the active payload, which is then transported across the lysosomal membrane into the cytoplasm or nucleus to induce cell death^1–4^. Several ADCs, such as gemtuzumab ozogamicin, brentuximab vedotin, and trastuzumab emtansine, have been approved for the treatment of acute myeloid leukaemia (AML), Hodgkin lymphoma, and HER2-positive breast cancer, respectively. Among these, trastuzumab emtansine is a standard second-line therapy for HER2-positive breast cancer^5^. Unfortunately, treatments with trastuzumab emtansine can still show disease relapses or progression due to the induction of drug resistance in patients. This drug resistance can be induced via different mechanisms, including (a) antibody-mediated resistance, (b) impaired drug trafficking, (c) disrupted lysosomal function, and (d) payload-related resistance^6^.

FMS-like tyrosine kinase 3 (FLT3), also known as CD135, is a type III receptor tyrosine kinase (RTK) expressed in early myeloid and lymphoid progenitor cells but not in erythroid, megakaryocytic, or macrophage lineages^7^^,^^8^. FLT3 receptors are significantly upregulated in AML blast cells and B-cell precursor acute lymphoblastic leukaemia (B-ALL) cells when compared to normal haematopoietic cells^9^^,^^10^. The FLT3 receptor plays a key role in haematopoietic stem/progenitor cell (HSPC) survival, proliferation, and differentiation^8^. Mutations in the FLT3 gene, particularly internal tandem duplications (FLT3-ITD), occur in approximately 20–27% of adult and 10–16% of paediatric AML cases and are associated with poor prognosis and high relapse rates^11^^,^^12^. Because of its overexpression in AML blasts, FLT3 protein receptor represents a promising molecular target for the selective delivery of cytotoxic drugs to leukemic cells.

Over the past decade, several FLT3 inhibitors have been developed to improve AML treatment outcomes. FLT3 inhibitors such as Sunitinib have been used as maintenance therapy after intensive chemotherapy^13^, while Midostaurin has been used alongside standard chemotherapy to significantly improve survival in patients with FLT3-ITD mutations^14^. Gilteritinib, a next-generation inhibitor, has been approved as monotherapy for relapsed or refractory FLT3-mutated AML and can reduce mortality risk by up to 36% compared with standard chemotherapy^15^. Unfortunately, although FLT3 inhibitors have been effective in clinical trials, they can generate drug resistance characteristics through innate and acquired resistance mechanisms^16^. The innate resistance mechanism involves several factors, including CYP3A4 upregulation, FGF2 secretion, FLT3 ligand activation, and CXCR4 expression; thus, one-third of patients exhibit innate resistance due to these factors^17–19^. Acquired resistance emerges over time from prolonged FLT3 inhibitors treatment through activation of alternative signalling pathways that allow cell survival despite FLT3 inhibition. The resistance is generated through new genetic alterations via clonal evolution, new mutations (e.g., TET2, RAS, IDH1/2), or activation of JAK/STAT5 and PI3K/AKT pathways^20–23^. Many researchers have explored various approaches to counteract resistance to FLT3 inhibitors with the goal of decreasing relapse rate as well as drug resistance in AML patients. To overcome drug resistance, several approaches have been explored, including developing novel FLT3 inhibitors and combining FLT3 inhibitors with standard chemotherapy^24^. There are also efforts to utilise FLT3 overexpression in AML blast cells to deliver cytotoxic drugs using ADCs and nanoparticles^25^^,^^26^. To be successful in using the FLT3 receptor for targeted drug delivery methods such as ADCs, these strategies require a deeper understanding of FLT3 receptor internalisation and intracellular trafficking mechanisms, which remain incompletely characterised.

In this study, we hypothesise that understanding receptor trafficking dynamics is essential for the rational design of FLT3-targeted ADCs. Receptor trafficking through the endosomal-lysosomal pathway plays a crucial role in releasing cytotoxic drugs from ADCs into the cytoplasm, where the drugs reach their targets and induce cell death. Impaired receptor trafficking or disrupted lysosomal function can hinder payload release, leading to reduced cytotoxicity and contributing to ADCs resistance. The binding and internalisation of FLT3 mAb were examined in FLT3-expressing (THP-1 and MV4-11) and FLT3-negative (K562) cells using flow cytometry and confocal microscopy. FLT3 mAb specifically bound to and was internalised into both FLT3-positive cells via receptor-mediated endocytosis, while no uptake occurred in FLT3-negative K562 cells. Confocal imaging showed distinct trafficking patterns in THP-1 (FLT3-wt) cells in which FLT3 mAb localised to lysosomes; whereas, in MV4-11 (FLT3-ITD) cells, it accumulated in the Golgi region. To evaluate the effect on ADCs efficacy, an FLT3-MMAE conjugate was synthesised, and its cytotoxicity was evaluated on FLT3-ITD MV4-11 cell and FLT3-wt THP-1 cells. We found that FLT3-MMAE conjugate showed higher cytotoxicity in THP-1 than in MV4-11 cells, despite similar binding properties. The decreased cytotoxicity of FLT3-MMAE conjugates in FLT3-ITD cells suggests impaired lysosomal delivery and inefficient payload release. Overall, these findings highlight that receptor trafficking differences contribute to ADCs resistance, and it emphasises the need to optimise lysosomal targeting or linker design for improved efficacy in FLT3-mutant AML.

## Materials and methods

### Cell culture and reagents

MV4-11 cells (CRL-9591^™^), KG-1a cells (CCL-246.1^™^), K562 cells (CCL-243^™^), THP-1 cells (TIB-202^™^) and U937 cells (CRL-1593.2^™^) were purchased from the American Type Culture Collection (Manassas, VA, US). EoL-1 cells (RBRC-RCB0641) were purchased from RIKEN BRC Cell Bank (Ibaraki, Japan).

MV4-11 cells were used as a representative model of leukemic cells harbouring the FLT3-ITD mutation. KG-1a cells, an acute myeloblastic leukaemia cell line enriched in stem cell populations, were also utilised. Both cell lines were cultured in Iscove’s Modified Dulbecco’s Medium (IMDM) supplemented with 20% foetal bovine serum (FBS), 100 U/mL penicillin, 100 µg/mL streptomycin, and 1.0 mM L-glutamine. K562 cells were used as an FLT3-negative control cell line. THP-1 cells and EoL-1 cells served as models of wild-type FLT3-overexpressing leukemic cells, while U937 cells represented acute monocytic leukaemia. K562, THP-1, EoL-1, and U937 cells were cultured in RPMI-1640 medium supplemented with 10% FBS, 100 U/mL penicillin, and 100 µg/mL streptomycin. All cell lines were maintained in a humidified incubator with 5% CO_2_ at 37 °C.

Iscove’s modified Dulbecco’s medium (IMDM), RPMI-1640 medium, penicillin-streptomycin, L-glutamine, Foetal bovine serum (FBS), Monensin, Brefeldin A, Hanks’ Balanced Salt Solution (HBSS), LysoTracker^™^ Red DND-99, 4% paraformaldehyde, permeabilization buffer, Alexa fluor 647-labelled GM130 polyclonal antibody (Golgi marker) and SiteClick^™^ Antibody Labelling Kits containing β‑Galactosidase, UDP‑GalNAz and β‑1,4‑galactosyltransferase (GalT) were purchased from Thermo Fisher Scientific (Waltham, MA, US). Sodium Azide (NaN_3_) and sucrose were purchased from Fisher Scientific (Waltham, MA, US). Bovine serum albumin (BSA), horse serum and 2-Deoxy-D-glucose were purchased from Sigma-Aldrich (St. Louis, MO, USA). Alexa fluor 488-labelled FLT3 mAb and anti-FLT3 receptor were purchased from Becton, Dickinson and Company (BD) (Chicago, IL, USA). PE-labelled FLT3 antibody was purchased from Biolegend (San Diego, CA, USA). Alexa fluor 647-labelled wheat germ agglutinin (WGA) and MTT were purchased from Biotium (Fremont, CA, USA). DAPI (4′,6-diamidino-2-phenylindole) was purchased from ApexBio Technology LLC (Houston, TX, USA). Antifade mounting media was purchased from Vector Laboratories (Newark, CA, USA). DBCO-PEG4-bal-cit-PAB-MMAE was purchased from Broadpharm (San Diego, CA, USA). Bafilomycin A1 (Baf A1) was purchased from MedChemExpress (Princeton, NJ, USA).

### Determination of surface FLT3 receptor expression on leukaemia cells

MV4-11, THP1, EOL-1, KG-1a, K562 and U937 cells were washed with PBS containing 0.1% (w/v) BSA three times followed by incubation with Fc block solution on ice. After 30 min, either Alexa fluor 488-labelled FLT3 antibody or PE-labelled FLT3 antibody was added and continuously incubated on ice in the dark for 1 h. Then, these cells were washed with cold PBS containing 0.1% (w/v) BSA twice followed by centrifugation to collect the cell pellet. Finally, Attune^™^ NxT Flow Cytometer (Thermo Fisher Scientific, Waltham, MA, USA) was used to analyse the stained cells.

### Antibody internalisation studies

After washing, MV4-11 cells were incubated with Fc block solution on ice for 30 min. For time-dependent binding and internalisation, 2 µg/mL of Alexa fluor 488-labelled FLT3 antibody (FLT3-AF488 mAb) was added and incubated for 30, 60, 120, 240, 360 min at 4 °C and 37 °C in the dark. For concentration-dependent binding and internalisation, cells were incubated with FLT3-AF488 mAb at 31.25, 62.5, 125, 250, 500, 1000 and 2000 ng/mL for 60 min in the dark at 4 °C and 37 °C. Cells were then washed twice with cold PBS + 0.1% (w/v) BSA and isolated by centrifugation. Finally, the stained cells were analysed through flow cytometry using the Attune^™^ NxT Flow Cytometer.

### Receptor-mediated endocytosis studies

MV4-11 cells were adjusted to 0.4 × 10^6^ cells/tube and incubated with Fc block solution for 30 min on an ice bath. After that, monensin at 2 µM and brefeldin A at 3.0 µg/mL were incubated for 30 min at 4 °C and 37 °C. Subsequently, 2 µg/mL of FLT3-AF488 mAb was added, followed by continuous incubation for 360 min in the dark at 4 °C and 37 °C. Then, cells were washed with cold PBS containing 0.1% (w/v) BSA by centrifugation. The stained cells were then analysed by flow cytometry using the Attune^™^ NxT Flow Cytometer.

### Energy depletion studies

Fc block solution was added to 0.4 × 10^6^ cells/tube of MV4-11 cells, followed by incubation for 30 min on ice. ATP depleting agents (i.e., 2.5 mM NaN_3_ and 1.5 mM 2-Deoxy-D-glucose) were added and incubated for 30 min at 4 °C and 37 °C. Next, 2 µg/mL of FLT3-AF488 mAb was added and continuously incubated at 4 °C and 37 °C in the dark. After 360 min, the cells were washed using cold PBS + 0.1% (w/v) BSA. Then, the stained cells were collected by centrifugation and analysed by flow cytometry using the Attune^™^ NxT Flow Cytometer.

### The intracellular trafficking of FLT3 mAb in FLT3-ITD and FLT3-wt cells

MV4-11 (FLT3-ITD), THP-1 (FLT3-wt) and K562 (FLT3-neg) cells were aliquoted into microfuge tubes at 5 × 10^5^ cells/sample in 50 µL of Fc block solution, and then, they were incubated for 30 min. The mixture was incubated with 2 µg/mL of FLT3-AF488 mAb for 60 min in the dark at either 4 °C or 37 °C. After washing the cells three times with HBSS containing 0.01% (w/v) sucrose, 500 nM of Lysotracker Red DND-99 (Lysosome marker) was added and incubated at 37 °C. After 45 min, the supernatant was removed, and the resulting cells were washed with HBSS containing 0.01% (w/v) sucrose three times. Next, 2% paraformaldehyde was used to fix the cells for 20 min at room temperature followed by washing with HBSS containing 0.01% (w/v) sucrose three times. 2 µg/mL of Alexa fluor 647-labelled wheat germ agglutinin (WGA) was incubated in cells for 45 min at room temperature followed by washing by HBSS containing 0.01% (w/v) sucrose (3 times). For Golgi staining, after the fixation step, the cells were permeabilized with 3% bovine serum albumin (BSA) in permeabilization buffer at room temperature for 10 min, followed by three washes with HBSS containing 0.01% (w/v) sucrose. The cells were then blocked with 2.5% horse serum in HBSS containing 0.01% (w/v) sucrose at room temperature for 30 min. Subsequently, 10 µg/mL of Alexa Fluor 647-labelled GM130 polyclonal antibody (Golgi marker) was added and incubated at room temperature for 60 min. To remove the excess of the antibody, the cells were washed three times with HBSS containing 0.01% (w/v) sucrose. For the last step, 1 µg/mL of DAPI was used to stain the nucleus upon DAPI incubation for 20 min at room temperature. The cell smear was mounted with antifade mounting media and visualised under a confocal microscope using a Leica Laser Scanning Confocal Upright Microscope (Model DM6-Q). Moreover, to confirm receptor-mediated endocytosis, monensin and brefeldin A were added and incubated for 30 min at 4 °C and 37 °C before adding Alexa 488-labelled FLT3 mAb (2 µg/mL). Also, energy depletion was confirmed using confocal microscopy. ATP depleting agent (i.e., 2.5 mM NaN_3_ or 1.5 mM 2-Deoxy-D-glucose) was added and incubated for 30 min at 4 °C and 37 °C before adding Alexa 488-labelled FLT3 mAb (2 µg/mL), lysosome staining, cell membrane staining, and nucleus staining, followed by cell fixation; then, microscopy observations were carried out in the manner described above.

### Site specific conjugation of MMAE to anti-FLT3-receptor mAb to make FLT3-MMAE conjugate

DBCO-PEG4-Val-cit-PAB-MMAE was conjugated with azide modified anti-FLT3-receptor mAb (FLT3-MMAE conjugate) via copper-free click chemistry following the SiteClick^™^ Antibody Labelling Kits procedure. Briefly, 1 mg of anti-FLT3-receptor mAb was concentrated using a protein concentrator with molecular weight cut-off (MWCO) 50 KDa. Next, the mAb was incubated with β-Galactosidase for 4 h at 37 °C. The azide modification solution was prepared using these components: 12 µL of dH_2_O, 18 μL of 20X Tris buffer, pH 7.0, and 40 μL of buffer additive. 160 μL of GalT enzyme was added into the tube containing UDP-GalNAz for 24 h at 37 °C. The excess UDP-GalNAz as a substrate was then removed with 1X Tris pH 7.0 using 50 KDa MWCO protein concentrator by centrifugation. After that, DBCO-PEG4-Val-cit-PAB-MMAE was added to the azide modified anti-FLT3-receptor mAb followed by incubation for 24 h at room temperature. The excess of DBCO-PEG4-Val-cit-PAB-MMAE were removed with 1X Tris pH 7.0 using 50 KDa MWCO protein concentrator by centrifugation. Finally, the conjugation of the FLT3-MMAE conjugate was collected and analysed using mass spectrometry. The FLT3-MMAE conjugate concentration was investigated by NanoDrop^™^ Onec Spectrophotometer (Thermo Fisher Scientific, Waltham, MA, USA).

### In vitro cytotoxicity of FLT3-MMAE conjugate on leukemic cell

MV4-11 (FLT3-ITD), THP-1 (FLT3-wt) and K562 (FLT3-neg) cells (5000 cells/100 µL) were incubated at 37 °C under 5% CO_2_ overnight; then, various concentrations (0.01, 0.1, 1, 10, 100 and 1000 nM) of MMAE, DBCO-PEG4-Val-cit-PAB-MMAE and FLT3-MMAE conjugate in 100 µL media were incubated in cell mixture for 96 h. Following 96 h of treatment, 100 µL of medium was removed and 15 µL (5 mg/mL) of MTT was then added and incubated for 4 h. Next, a solubilising reagent (200 µL of DMSO) was added and mixed well. The resulting purple solution in a microplate reader was measured at 578 nm along with the reference wavelength at 630 nm. The % cell viability was determined using the absorbance of test sample and vehicle control wells by utilising the following equation:

% Cell viability =[Absorbance of test x100]/[Absorbance of vehicle control].

The percentage of cell viability was plotted as a concentration response curve to determine the 50% inhibition of viability (IC_50_) of MMAE, DBCO-PEG4-Val-cit-PAB-MMAE and FLT3-MMAE conjugate.

### Lysosomal activity studies

MV4-11 and THP1 cells were added into 96-well plates at 5000 cells/100 µL/well. 0.01, 0.1 and 1 nM of Bafilomycin A1 were then added followed by incubation for 1 h. After that, the cells were incubated with 1000 nM of FLT3-MMAE conjugate for 92 h. Finally, the cell viability was determined by MTT assay as described above.

## Result

### Determination of FLT3 receptors on the surface of leukaemia cells

The expression of the FLT3 receptor on leukemic cell lines, including MV4-11 (FLT3-ITD), THP-1 (FLT3-wt), EOL-1 (FLT3-wt), KG-1a (leukemic stem-like cells), K562 (FLT3-negative), and U937 (FLT3-negative), was determined by staining with PE-labelled anti-FLT3 monoclonal antibodies (mAb). Fluorescence intensities were measured by flow cytometry. Antibody staining revealed that only MV4-11, THP-1 and EOL-1 cells expressed the FLT3 receptor on the cell surface, while no FLT3 receptor expression was detected on the cell surface of KG-1a, K562, and U937 cells (Supplementary Figure S1).

**Figure 1.**
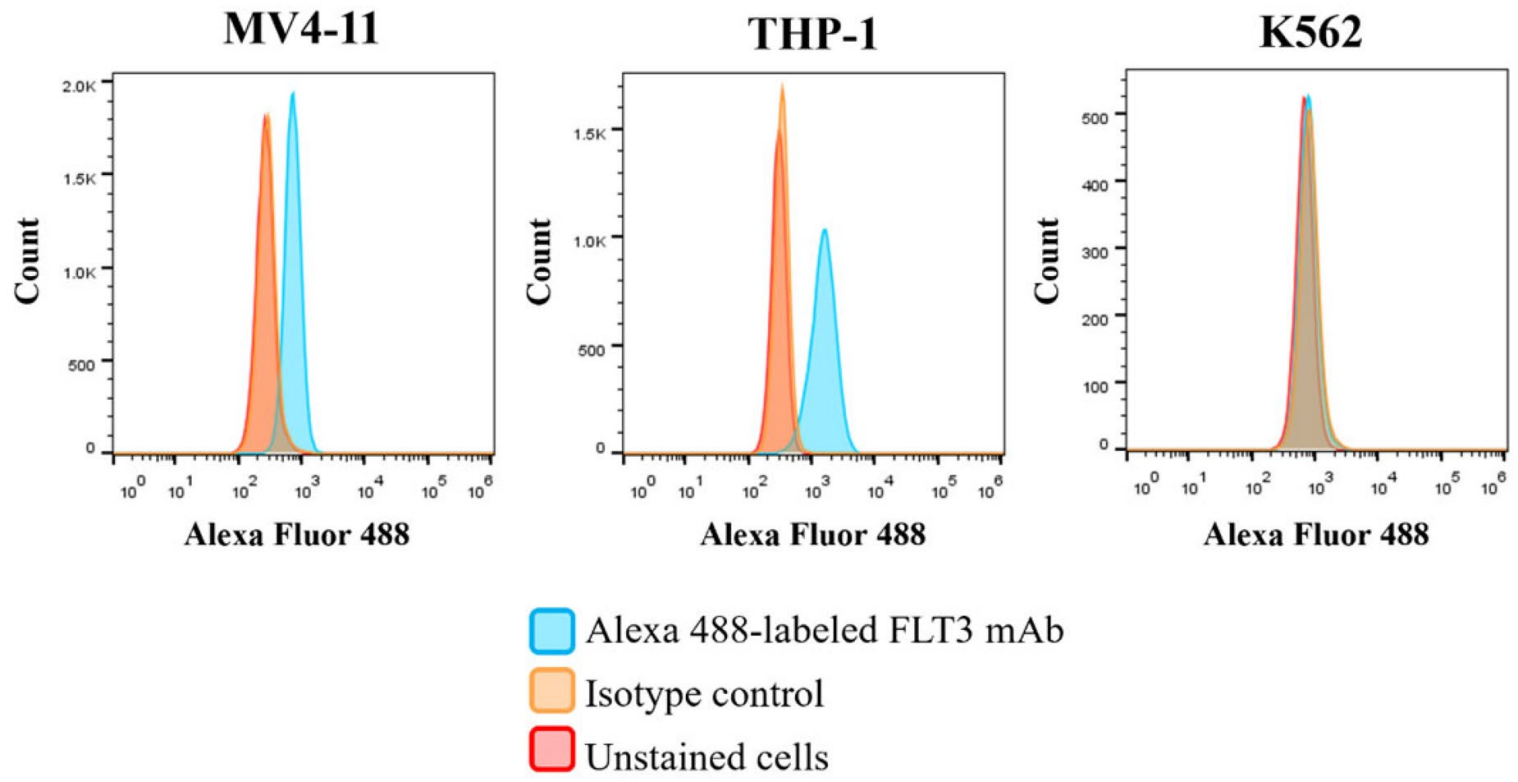
The surface expression of FLT3 receptor in MV4-11 (FLT3-ITD) and THP-1 (FLT3-wt) cells compared to K562 (FLT3-neg). Theexpression of FLT3 on the surface of both cells was determined by flow cytometry. The histograms showed FLT3 mAb bound to FLT3 receptors on both mutant and wild cell lines.

Moreover, surface expressions of the FLT3 receptor in MV4-11 (FLT3-ITD), THP-1(FLT3-wt) cells and K562 (FLT3-negative) were further confirmed using Alexa Fluor 488-labelled anti-FLT3 mAb (FLT3 mAb). In this study, MV4-11 cells (human acute myelomonocytic leukaemia) were used as the FLT3-ITD mutated cell line, whereas THP-1 cells (human acute monocytic leukaemia) were used as the FLT3 wild-type (FLT3-wt) cell line and K562 (chronic myeloid leukaemia) represent as FLT3 negative cell line. The results showed significantly higher fluorescence intensities in both FLT3-ITD-mutated MV4-11 and FLT3-wt THP-1 cells compared with FLT3-neg K562 cells (Figure 1). These findings confirmed the expression of FLT3 receptors on both mutant and wild type cell lines.

### Internalisation of FLT3 mAb by FLT3 receptor-mediated endocytosis on leukaemia cells

**Figure 2.**
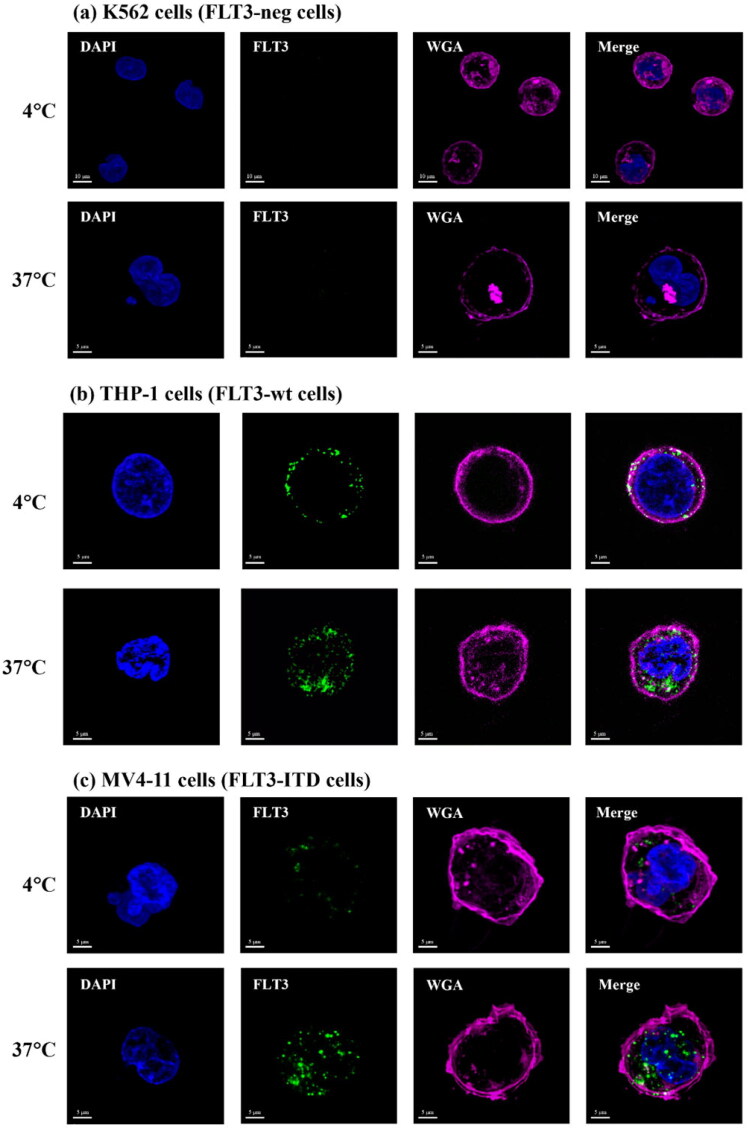
Temperature-dependent internalisation of FLT3 mAb in different FLT3-expressing cells. Confocal fluorescence images showing FLT3 mAb (green) localisation in (a) K562 (FLT3-negative), (b) THP-1 (FLT3-wt), and (c) MV4-11 (FLT3-ITD) cells incubated at 4 °C or 37 °C. Nuclei were stained with DAPI (blue), and plasma membranes were labelled with WGA (magenta). No fluorescent signal of FLT3 mAb was observed in K562 cells at either temperature, while both THP-1 and MV4-11 cells showed strong fluorescent accumulation of FLT3 mAb inside the cells at 37 °C, indicating temperature-dependent internalisation through receptor-mediated endocytosis.

The binding and internalisation of FLT3-AF488 mAb to FLT3 receptors on K562 (FLT3-Neg), THP-1 (FLT3-wt) and MV4-11 (FLT3-ITD) cells were observed using confocal microscopy. The cells were incubated with FLT3-AF488 mAb (green) at 4 °C to assess antibody binding and at 37 °C to evaluate antibody internalisation. Cellular organelles were stained with Alexa Fluor 647-conjugated wheat germ agglutinin (WGA, magenta) to label the plasma membrane and with DAPI (blue) to stain the nuclei. As shown in Figure 2, no uptake of FLT3-AF488 mAb was observed in K562 cells (Figure 2a) at either 4 °C or 37 °C, confirming the absence of FLT3 receptor expression on their surface while both THP-1 (FLT3-wt) and MV4-11 (FLT3-ITD) cells exhibited stronger fluorescence at 37 °C than at 4 °C (Figure 2b-c).

**Figure 3.**
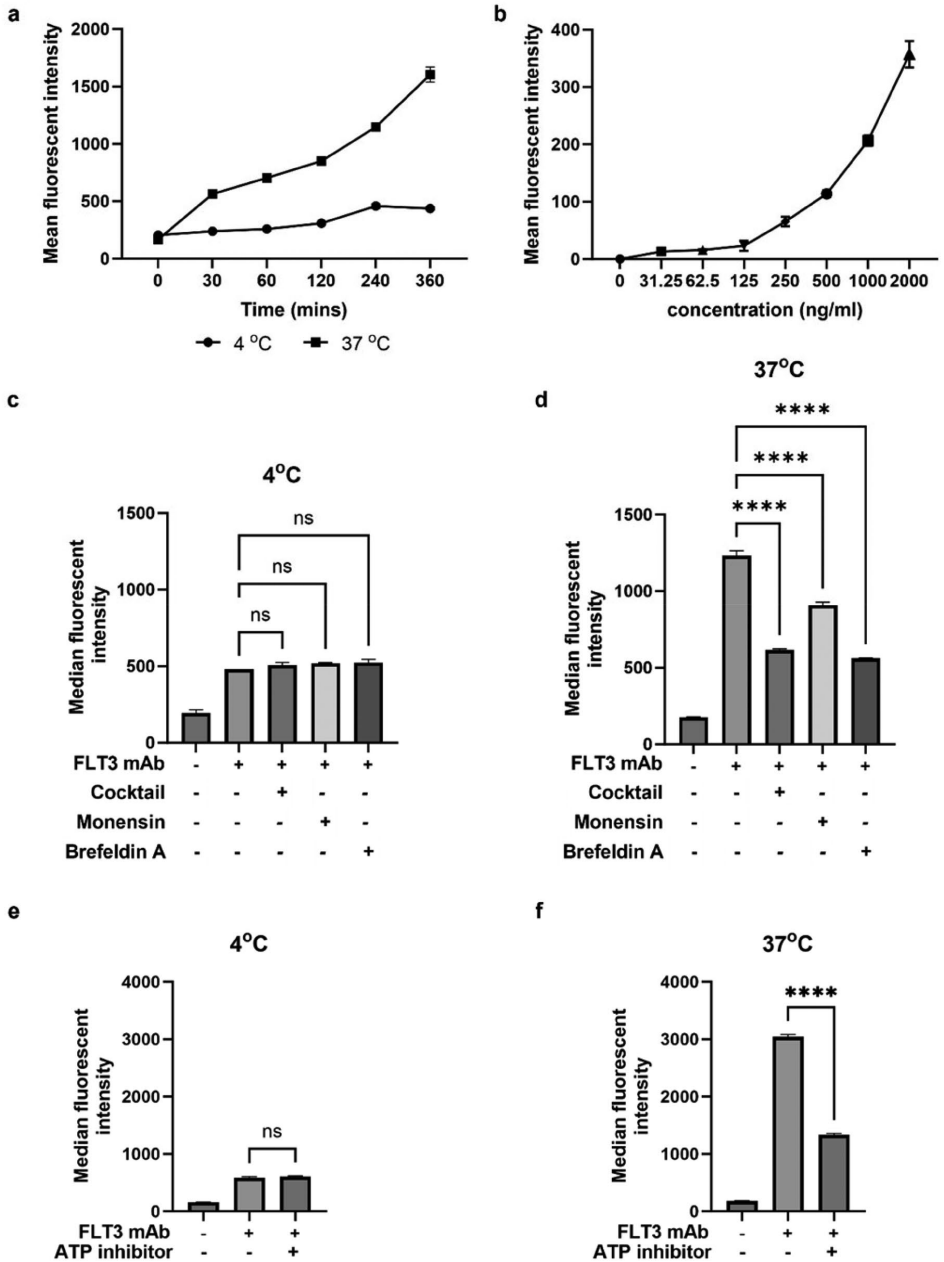
The effects of various conditions on binding and internalisation of FLT3 mAb by FLT3 receptors on MV4-11 cells as observed using flow cytometry. (a) The incubation time influences the uptake of mAb into MV4-11 cells at 37 °C, while the incubation time did not affect the cellular uptake at 4 °C. (b) The effects of various concentrations of FLT3 mAb on FLT3 receptor binding were evaluated at 4 °C. (c) The receptor recycling inhibitors such as monensin, brefeldin A, and the cocktail did not have any effect on FLT3 internalisation 4 °C. (d) Monensin, brefeldin A, and the cocktail significantly inhibited the internalisation of FLT3 receptors at 37 °C. (e) ATP depleting agents (NaN_3_ and 2-Deoxy-D-glucose) did not have any impact on the internalisation of the receptors at 4 °C. (f) NaN_3_ and 2-Deoxy-D-glucose as ATP inhibitors significantly suppress the uptake of FLT3 mAb into MV4-11 cells at 37 °C. The data showed mean ± SD in three independent experiments. The statistical differences were determined using one-way ANOVA and were considered significant at ****p < 0.0001.

Moreover, time- and temperature-dependent internalisation of the antibody via the FLT3 receptor in FLT3-expressing MV4-11 cells was confirmed by flow cytometry. The results demonstrated that the fluorescence intensity of FLT3-AF488 mAb increased in a time-dependent manner at 37 °C, whereas there was a limited increase in fluorescent intensity at 4 °C, where internalisation was inhibited (Figure 3a). These findings confirm that FLT3-AF488 mAb is internalised into cells through the FLT3 receptor on the cell surface in a temperature-dependent manner.

In addition, the effects of various concentrations of FLT3-AF488 mAb on FLT3 receptor binding were evaluated at 4 °C using flow cytometry (Figure 3b). The fluorescence intensity of FLT3-AF488 mAb increased in a concentration-dependent manner, indicating concentration-dependent receptor binding on the surface of MV4-11 cells. Because the experiment was performed at 4 °C, where endocytosis is inhibited, the observed fluorescence increase reflects specific receptor-antibody interactions on the cell membrane rather than internalised antibody. These results suggest that FLT3-AF488 mAb binding to the FLT3 receptor follows a receptor-mediated endocytosis profile.

**Figure 4.**
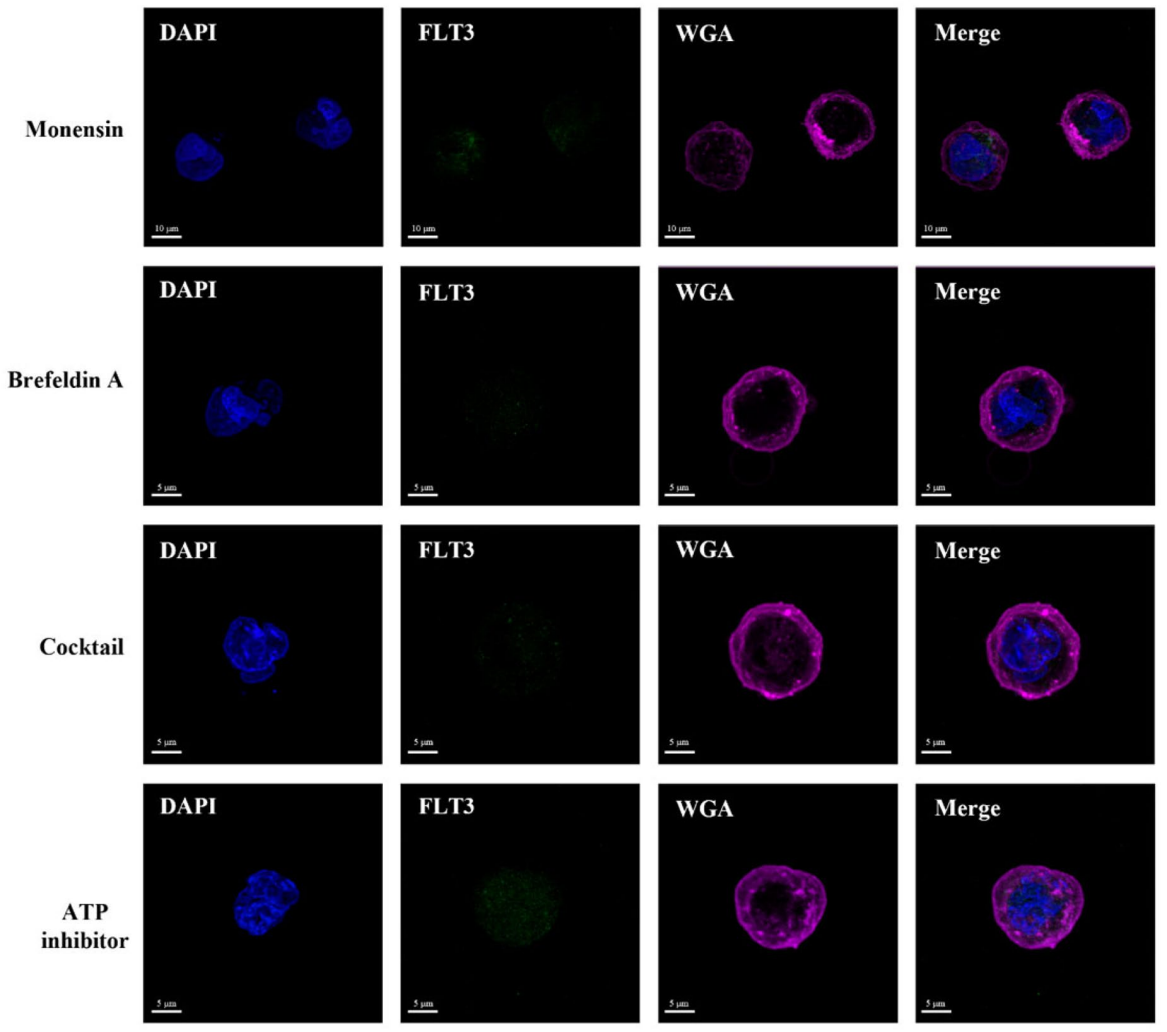
Effect of endocytosis inhibitors on FLT3 mAb internalisation. Confocal fluorescence images showing FLT3 mAb (green) localisation in cells treated with endocytosis inhibitors including Monensin, Brefeldin A, an endocytosis inhibitor cocktail, or an ATP inhibitor (NaN_3_ and 2- deoxy-D-glucose) at 37 °C. Nuclei were stained with DAPI (blue), and plasma membranes were labelled with WGA (magenta). Pre-treatment with endocytosis inhibitors blocked FLT3 mAb internalisation, indicating that FLT3 mAb uptake occurs through receptor-mediated endocytosis.

To confirm that FLT3-AF488 mAb internalisation occurred via a receptor-mediated endocytic pathway, the recycling of FLT3 receptors was inhibited using two endocytosis inhibitors: monensin and brefeldin A, either individually or in combination (cocktail). Monensin is an ionophoric antibiotic that can inhibit receptor endocytosis by selectively complexing with sodium cation for its transport across lipid membranes. It has been shown to inhibit the endocytic trafficking of peptide transporters responsible for shuttling large peptides across cell membranes^27^. Brefeldin A, a fungal metabolite, is widely used to study membrane trafficking as it blocks early stages of receptor-mediated endocytosis by reducing the formation of clathrin-coated pits and vesicles, thus impairing protein transport^28^. In this experiment, MV4-11 cells were preincubated with the inhibitors, followed by treatment with FLT3-AF488 mAb. Fluorescence intensities were analysed by flow cytometry and confocal microscopy. As expected, the inhibitors did not affect FLT3-AF488 mAb binding at 4 °C, where cellular uptake is already inhibited (Figure 3c). In contrast, at 37 °C, the internalisation of FLT3-AF488 mAb was suppressed in the presence of monensin, brefeldin A, or their combination (cocktail) (Figure 3d). Confocal imaging studies further confirmed that there was no internalisation of FLT3-AF488 mAb under these inhibitory conditions (Figure 4). These results indicate that the internalisation of FLT3-AF488 mAb in MV4-11 cells occurs through the FLT3 receptor-mediated endocytosis.

Energy dependence of FLT3-AF488 mAb internalisation was further examined using ATP depletion inhibitors, as receptor-mediated endocytosis is an energy-dependent process. Two metabolic inhibitors, 2-deoxy-D-glucose (2-DG) and sodium azide (NaN_3_), were used to block ATP production. 2-DG, a glucose analog, inhibits glycolysis by blocking the activity of hexokinase and glucose-6-phosphate isomerase, thereby preventing ATP generation through the glycolytic pathway^29^. NaN_3_ disrupts oxidative phosphorylation by impeding cytochrome oxidase, which is the terminal enzyme in the mitochondrial electron transport chain. As a consequence, NaN_3_ reduces the intracellular levels of ATP^30^. In this study, MV4-11 cells were preincubated with 2-DG and NaN_3_ before incubating to FLT3-AF488 mAb. The fluorescence intensities were subsequently determined by flow cytometry and confocal microscopy. As expected, ATP inhibition had no effect on FLT3-AF488 mAb binding at 4 °C, where endocytosis is blocked (Figure 3e). However, at 37 °C, there was a significant difference between the cells pre-treated in the presence of ATP inhibitors compared with cells treated with the antibody alone (Figure 3f). The confocal imaging study also showed no internalisation of FLT3-AF488 mAb in the presence of ATP inhibitors (Figure 4). These results confirmed that the major route of entry of FLT3-AF488 mAb into MV4-11 cells is via the receptor-mediated endocytic process.

### Differential subcellular localisation of FLT3-ITD and FLT3-wt in leukaemia cells

To determine whether the intracellular trafficking of FLT3 mAb in FLT3-ITD and FLT3-wt cells involves the endosomal-lysosomal pathway, MV4-11 (FLT3-ITD) and THP-1 (FLT3-wt) cells were incubated with FLT3-AF488 mAb (green) at 37 °C for 1 h. Next, Lysosomes were stained with LysoTracker^™^ (red), and the Golgi apparatus was labelled with GM130 antibody (magenta). The stained cells were then visualised using confocal microscopy.

**Figure 5.**
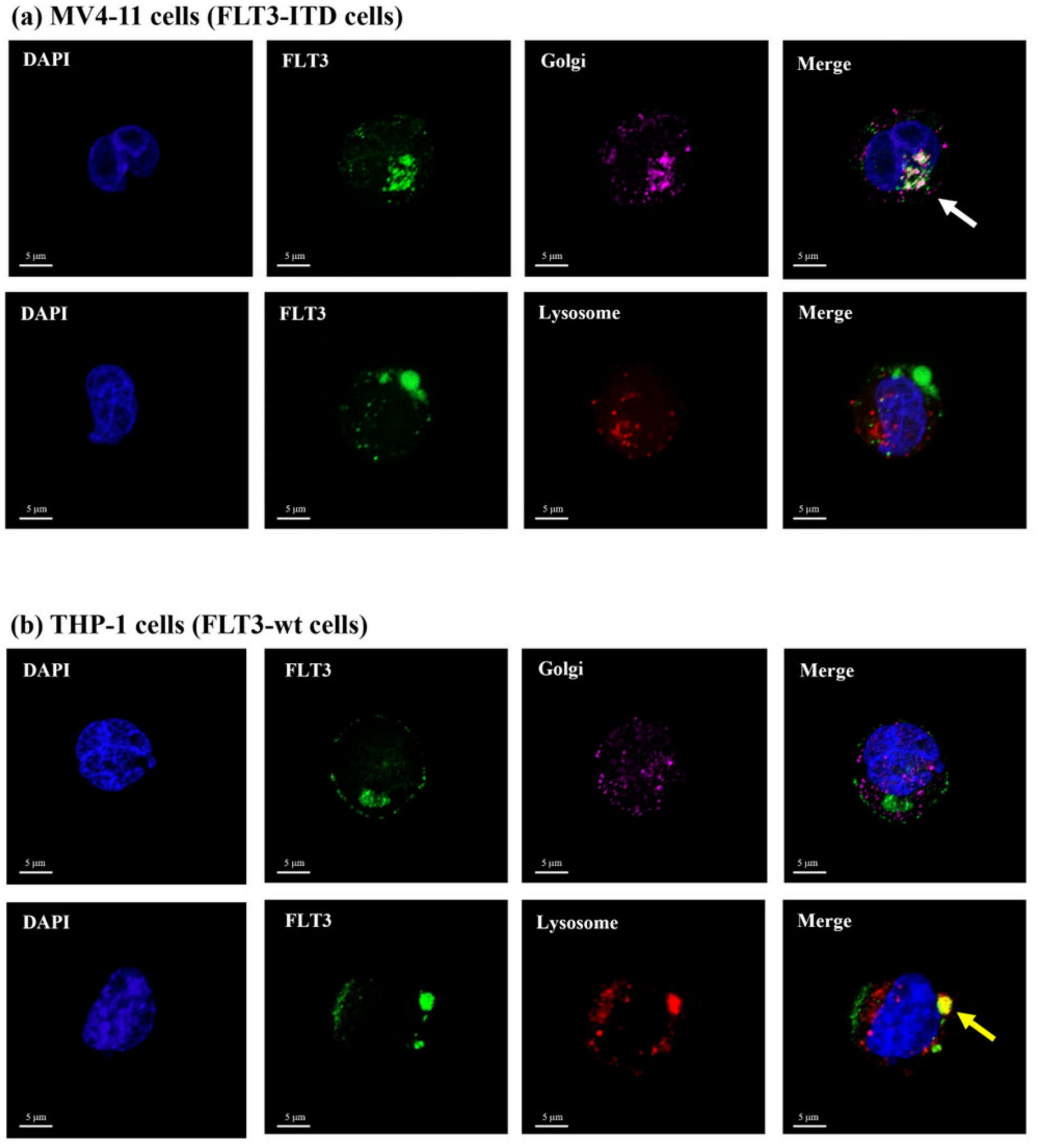
Differential localisation pathways of FLT3 mAb (green) in (a) FLT3-ITD MV4-11 and (b) FLT3-wt THP-1 cells. The cells were incubated with FLT3-AF488 mAb (green) at 37 °C for 1 h. Cellular organelles were stained with LysoTracker^™^ (red, lysosomal marker), GM130 (magenta, Golgi marker), and DAPI (blue, nuclear marker). In MV4-11 cells, FLT3 predominantly co-localised with the Golgi marker (white arrow), indicating accumulation in the Golgi region. In contrast, THP-1 cells showed strong co-localisation of FLT3 with the lysosomal marker (yellow arrow), suggesting normal receptor trafficking through the endosomal-lysosomal pathway.

As shown in Figure 5(a), no colocalization was observed between FLT3-AF488 mAb (green) and LysoTracker (red) in MV4-11 cells, indicating that FLT3-ITD does not traffic through the endosomal-lysosomal pathway. Instead, strong colocalization was detected between FLT3-AF488 mAb and the Golgi marker GM130 (magenta), suggesting that FLT3-ITD is predominantly localised to the Golgi apparatus rather than lysosomes. In contrast, THP-1 (FLT3-wt) cells exhibited significant colocalization between FLT3-AF488 mAb and LysoTracker (red), but not with GM130 (magenta), indicating that the FLT3-wt receptor is internalised via the endosomal-lysosomal trafficking pathway (Figure 5b). These results indicate that the FLT3-ITD mutation disrupts normal receptor trafficking, resulting in FLT3 mAb accumulation in the perinuclear Golgi region.

### Synthesis and characterisation FLT3-MMAE conjugate

Since the FLT3-ITD mutation alters normal receptor trafficking and causes FLT3 mAb to accumulate in the perinuclear Golgi region, we investigated whether this mislocalization affects the efficacy of antibody drug conjugates (ADCs). Specifically, we examined whether the absence of lysosomal trafficking and consequently, the lack of enzymatic degradation could impair payload drug release and reduce ADCs potency.

**Figure 6.**
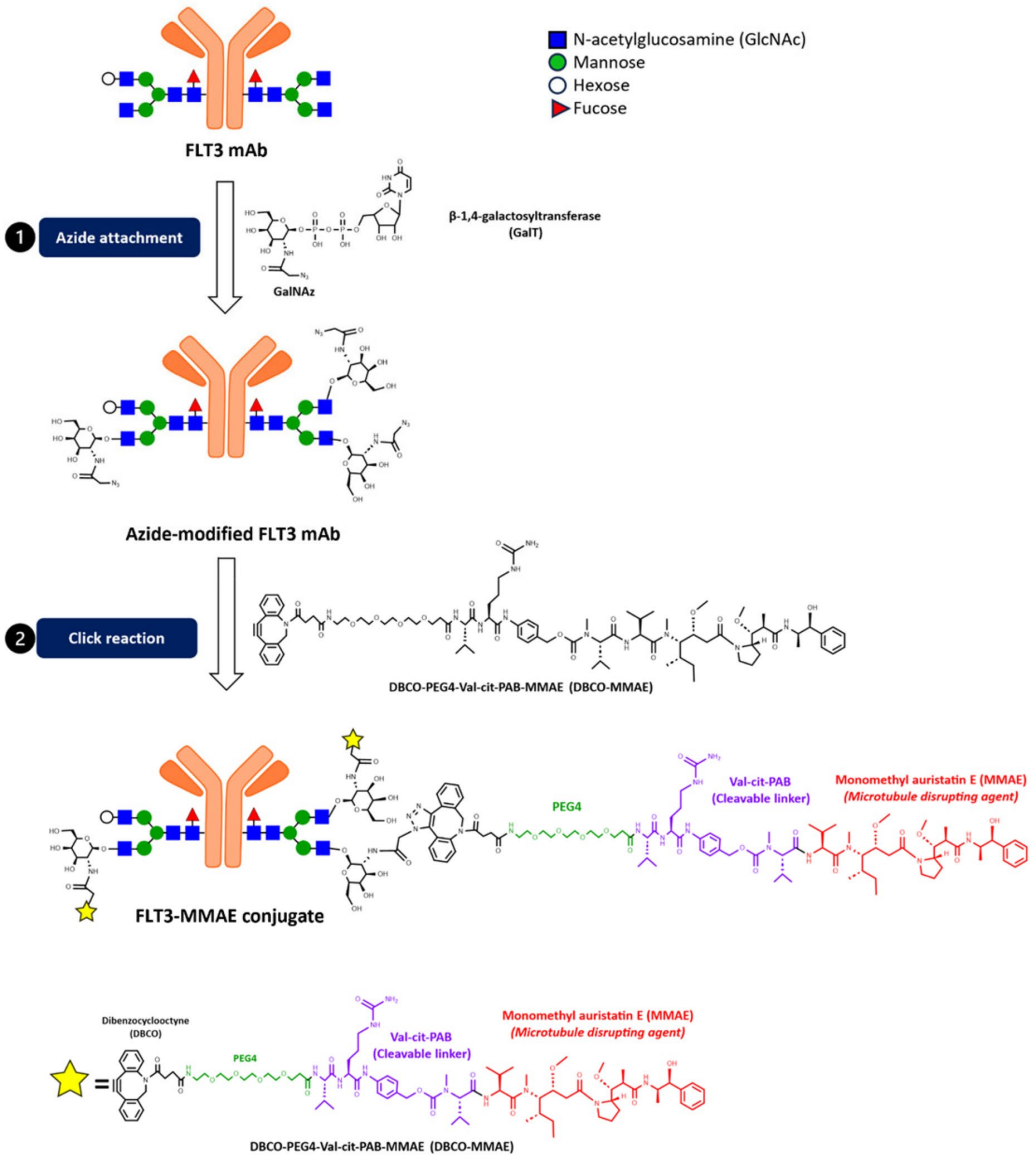
Synthetic scheme for the preparation of the FLT3-MMAE conjugate as ADC. The FLT3-MMAE conjugate was synthesised by conjugating the FLT3 mAb to the cytotoxic payload MMAE using copper-free click chemistry. The FLT3 mAb was first modified by adding the reactive azide group via conjugation of GalNAz to the terminal GlcNAc residues on the Fc N-glycans using β-1,4-galactosyltransferase (GalT). The MMAE cytotoxic agent was then attached by reacting the azide-functionalized antibody with DBCO-PEG4-Val-Cit-PAB-MMAE via copper-free click chemistry to produce the desired FLT3-MMAE conjugate. The maximum theoretical conjugation stoichiometry is *n* = 3, based on the Fc glycan composition.

Therefore, we synthesised an ADCs called FLT3-MMAE conjugate to evaluate this idea (Figure 6). The FLT3-MMAE conjugate contains monomethyl auristatin E (MMAE) as the drug payload, which is a potent anti-cancer drug that targets microtubules. As the payload, MMAE is one of the most used payloads in ADCs under clinical development. It is a highly potent antimitotic agent with cytotoxicity 100–1000 times greater than doxorubicin; however, its clinical use has been limited due to severe toxicity to normal cells. ADCs can solve this issue through a prodrug strategy, enhancing tumour selectivity via antibody-mediated targeting of overexpressed receptors in tumour cells. MMAE induces G2/M-phase growth arrest by binding to tubulin dimers, which blocks tubulin polymerisation and disrupts the mitotic spindle formation in nuclear division. The subsequent mitotic arrest eventually leads to cell death via apoptosis induction^31^^,^^32^. The MMAE was conjugated to the *N-*glycan site on FLT3 mAb via the cleavable valine-citrulline-p-aminobenzyloxycarbonyl (Val-Cit-PAB) linker. This drug can be cleaved from the linker via enzymatic reaction by cathepsin B in the lysosomes^33^^,^^34^. ADCs bearing Val-Cit-PAB linkers are designed to undergo proteolytic activation predominantly within lysosomes, where cathepsin B is highly active under acidic conditions. In contrast, the Golgi apparatus lacks sufficient cathepsin B activity and appropriate proteolytic conditions, making it an unfavourable compartment for linker cleavage and payload release^35^.

**Figure 7.**
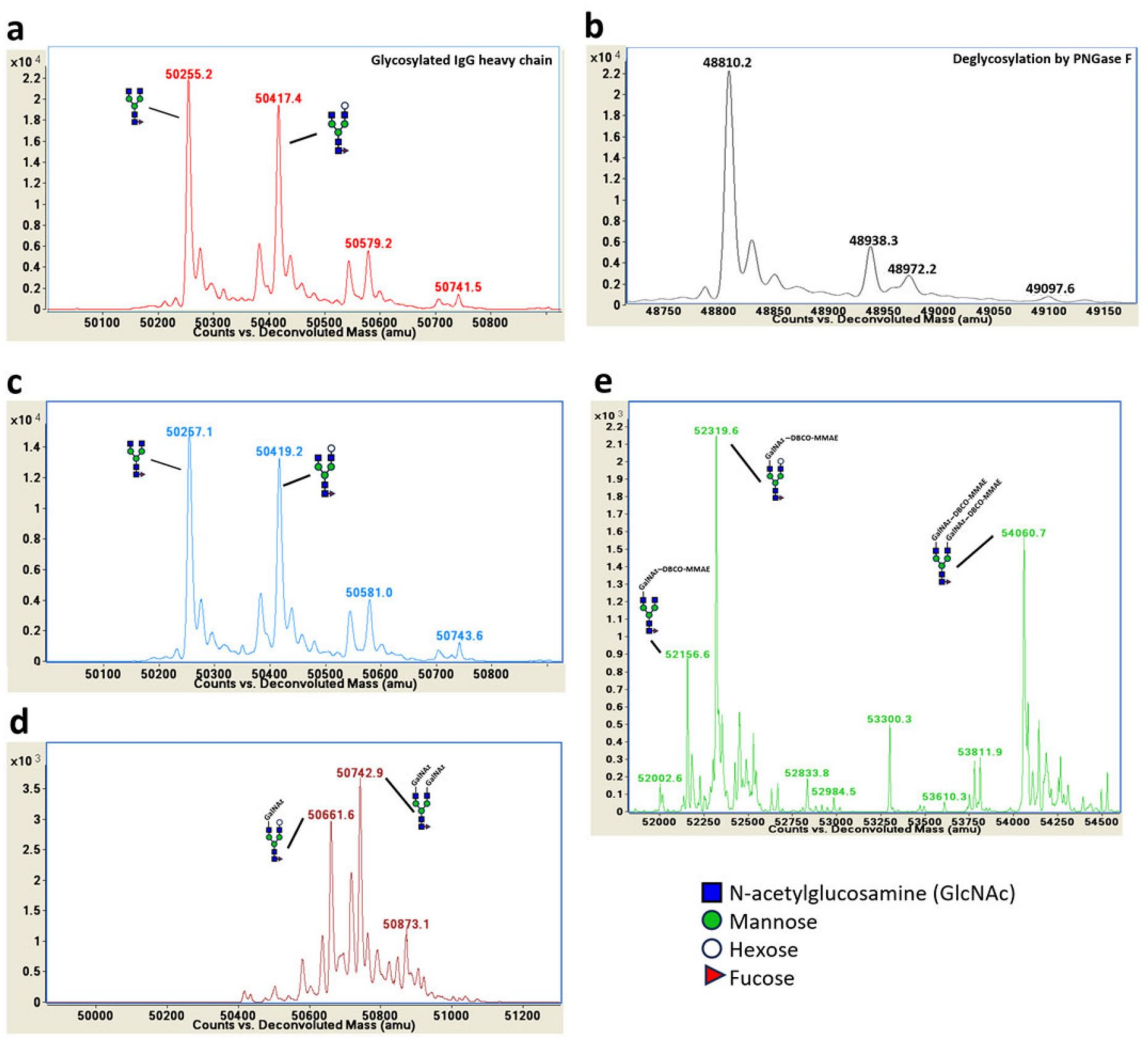
The ESI-QTOF mass spectrometry analysis of FLT3 mAb and its derivatives: (a) parent FLT3 mAb, (b) FLT3 mAb after treatment with PNGase F, (c) FLT3 mAb after treatment with β-1,4 Galactosidase, (d) FLT3 mAb after reaction with GalNAz, and (e) a final desired product, FLT3-MMAE conjugate.

The product of each step was characterised by ESI-QTOF mass spectrometry (Figure 7). To determine the main glycan structure of FLT3 mAb, its glycan structure was characterised using ESI-QTOF mass spectrometry. As shown in Figure 7(a), there were two major peaks for the heavy chain at 50255.2 Da and 50417 Da; this is due to the difference in glycosylation states of the mAb. Next, FLT3 mAb was treated with PNGase F to remove N-glycans. The reaction revealed a major peak at 48810.2 Da corresponding to the molecular weight of the deglycosylated mAb heavy chain with C-terminal lysine removed (Figure 7b). A minor peak at 48938.3 Da corresponds to the molecular weight of the deglycosylated mAb heavy chain with C-terminal lysine intact (Figure 7b). The 1445 Da difference between the major form of the deglycosylated mAb heavy chain and the parent glycosylated mAb heavy chain is consistent with the presence of the G0F glycoform as the predominant N-linked glycan on the FLT3 mAb. The 1607 Da difference observed for the second most abundant glycoform of the parent mAb corresponds to the G1F glycoform or other N-linked glycoform isomers with identical mass. The terminal galactose of this second glycan might be linked through an α1,3 linkage, which is found in non-primate mammalian and is not cleaved by β1,4-galactosidase. However, peptide mapping of the FLT3 mAb was not performed to confirm the specific glycoform structure in this study.

The synthesis of FLT3-MMAE conjugate was started by reacting the GalNAz with the azide moiety to the galactose group on the FLT3 mAb via enzymatic reaction using GalT enzyme (Figure 6). To achieve this, we attempted to remove the additional hexose residue from the glycoform corresponding to the 50417 Da peak using β1,4-galactosidase (Figure 7c). Unfortunately, the terminal galactose of this glycan could not be cleaved by β1,4-Galactosidase, likely due to the reason described earlier.

The GlcNAc moieties in each FLT3 mAb molecule were reacted with UDP-GalNAz in the presence of β-1,4-galactosyltransferase (Gal-T) enzyme to transfer GalNAz to the terminal of non-reducing GlcNAc residues of the heavy chain (Figure 7d). The 244.2 Da mass difference between the 50,417.4 Da peak (corresponding to the G1F glycoform or other N-linked glycoform isomers with identical mass) and the 50,661.6 Da peak corresponds to the addition of one GalNAz unit to this glycoform. Moreover, the 487.8 Da mass difference between the G0F glycoform and the 50,742.9 Da peak corresponds to the addition of two GalNAz units to the G0F glycoform (Figure 7d). Finally, the azide groups of GalNAz-modified FLT3 mAb were reacted with DBCO-PEG4-Val-cit-PAB-MMAE (DBCO-MMAE) using the Click reaction. We identified three products. The first product, with MW of 52,156.6 Da, corresponded to the FLT3 mAb with one moiety of DBCO-MMAE conjugated to G0F. The second product had an MW of 52,319.6 Da, where one moiety of DBCO-MMAE was conjugated to the second glycan on the FLT3 mAb. Finally, the FLT3 mAb containing two moieties of DBCO-MMAE conjugated at G0F had an MW of 54,060.7 Da **(**Figure 7e**)**.

### In vitro cytotoxicity of FLT3-MMAE conjugate on FLT3 expressing leukemic cell

The cytotoxicity of the FLT3-MMAE conjugate was evaluated using an MTT assay in three leukaemia cell lines to determine whether impaired lysosomal trafficking of the FLT3-ITD receptor and reduced enzymatic degradation limit payload release and cytotoxicity.

**Figure 8.**
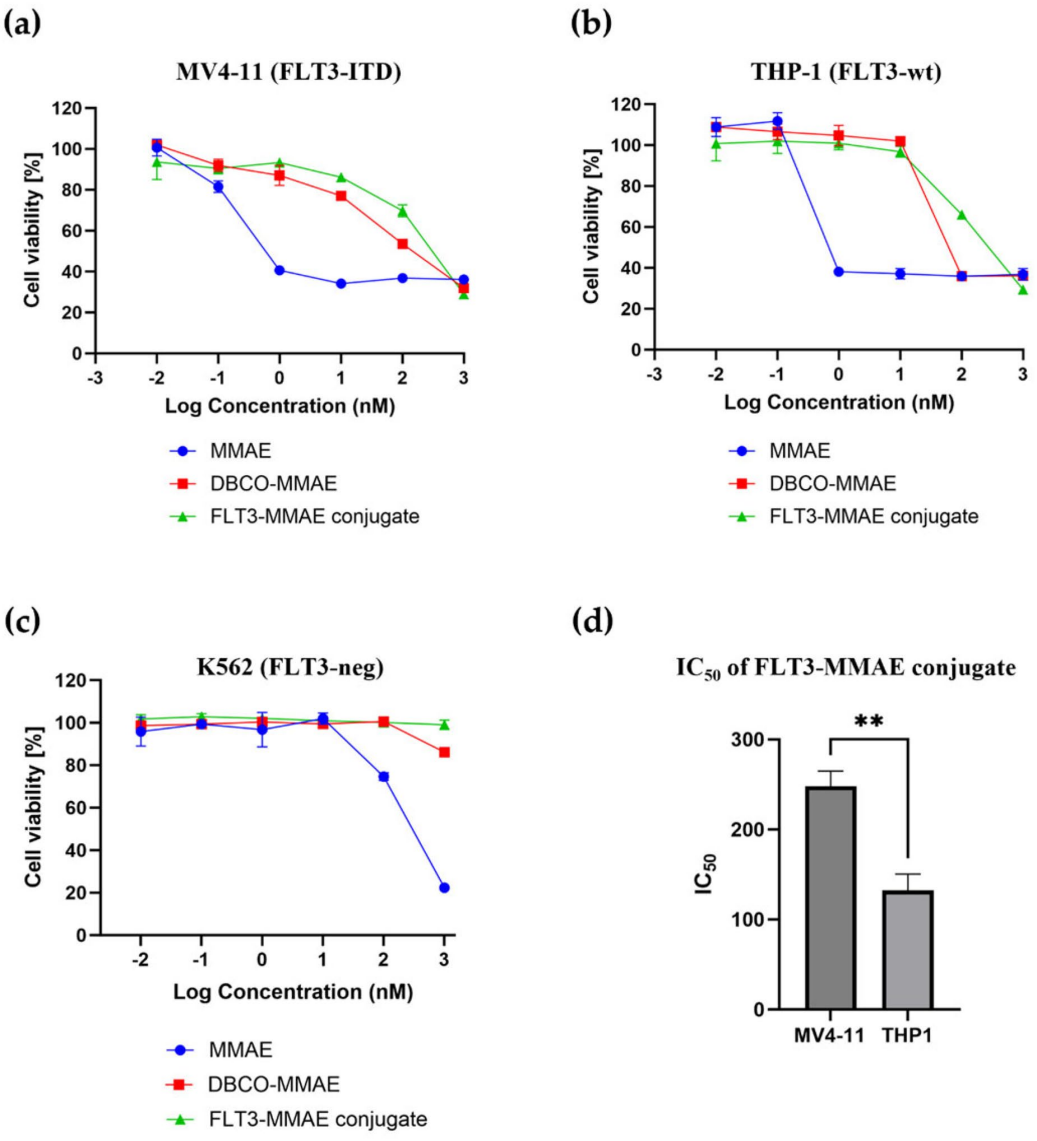
Cytotoxicity of FLT3-MMAE conjugate (green), DBCO-MMAE (red) and MMAE (blue) in (a) MV4-11 (FLT3-ITD), (b) THP-1 (FLT3-wt), and (c) K562 (FLT3-neg) cells after 96 h incubation. Cell viability was determined using the MTT assay. (d) Comparison of IC_50_ values of the FLT3-MMAE conjugate in MV4-11 and THP-1 cells, indicating significantly higher cytotoxicity in FLT3-wt THP-1 cells compared with FLT3-ITD MV4-11 (*p* < 0.01). The data represented mean ± SD from triplicate independent experiments.

MV4-11 (FLT3-ITD) cells were used to represent disrupted lysosomal trafficking via the FLT3-ITD receptor (Figure 5a) while THP-1 (FLT3-wt) cells represented normal receptor trafficking into lysosomes (Figure 5b). Finally, K562 cells served as a negative control lacking FLT3 receptor expression. The cytotoxic effect of the FLT3-MMAE conjugate was compared to those of DBCO-PEG4-Val-cit-PAB-MMAE (DBCO-MMAE) and free MMAE. As shown in Figure 8, FLT3-MMAE conjugate exhibited potent and dose-dependent cytotoxicity in both FLT3-expressing cells (MV4-11 and THP-1 cells), whereas little or no cytotoxic effect was observed in the FLT3-negative K562 cells. The IC_50_ values of MMAE, DBCO-MMAE, and FLT3-MMAE conjugate in MV4-11 cells were 0.17 ± 0.03, 40.73 ± 6.04 and 247.93 ± 17.00 nM, respectively (Table 1). The IC_50_ values of MMAE, DBCO-MMAE, and FLT3-MMAE conjugate in THP-1 cells were 0.29 ± 0.02, 32.89 ± 1.64 and 132.10 ± 18.29 nM, respectively (Table 1). Interestingly, IC_50_ values of FLT3-MMAE conjugate in MV4-11 (FLT3-ITD) cells were significantly higher than those in THP-1 (FLT3-wt) cells (*p* < 0.01) (Figure 8d), indicating that the FLT3-MMAE conjugate exhibited significantly stronger cytotoxic effects in THP-1 cells compared with MV4-11 cells.

**Table 1.** The IC_50_ (nM) values of MMAE, DBCO-MMAE and FLT3-MMAE conjugate in MV4-11 (FLT3-ITD) and THP-1 (FLT3-wt) cells.

Sample	MV4-11 (FLT3-ITD)	THP-1 (FLT3-wt)
**MMAE**	0.17 ± 0.03	0.29 ± 0.02
**DBCO-MMAE**	40.73 ± 6.04	32.89 ± 1.64
**FLT3-MMAE conjugate**	247.93 ± 17.00	132.10 ± 18.29

This observation confirmed that intracellular trafficking and processing of the conjugate differed between the two cell types: FLT3-ITD MV4-11 and FLT3-wt THP-1 cells. Previous confocal microscopy studies revealed that in THP-1 (FLT3-wt) cells (Figure 5b), the FLT3-AF488 efficiently trafficked to lysosomes, enabling effective payload release. In contrast, in MV4-11 cells harbouring the FLT3-ITD mutation, the conjugate accumulated predominantly in the perinuclear Golgi region (Figure 5a). Based on these observations, we inferred that the FLT3–MMAE conjugate fails to reach lysosomes in FLT3-ITD cells, thereby limiting lysosomal degradation and reducing MMAE release.

These findings highlight the influence of FLT3 intracellular localisation on ADCs efficacy, where the altered trafficking pattern in FLT3-ITD cells may compromise the cytotoxic activity of the conjugate and highlight the critical role of the endosomal-lysosomal trafficking pathway in determining the therapeutic efficacy of antibody drug conjugates. Overall, the results suggest that the therapeutic efficiency of FLT3-targeted ADCs depends not only on receptor-mediated internalisation but also on endosomal-lysosomal trafficking pathway.

### Lysosomal activity studies

**Figure 9.**
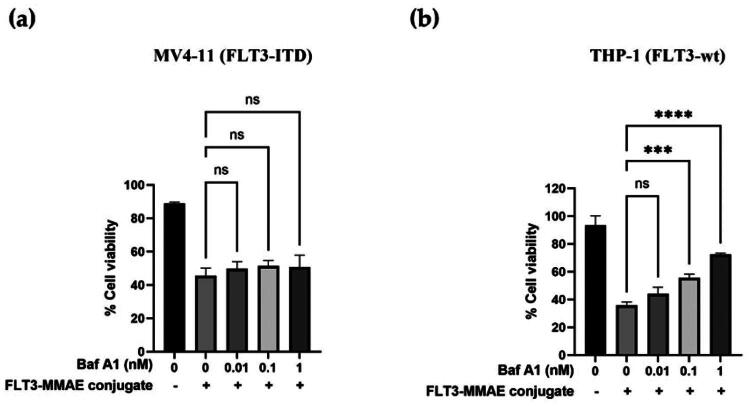
Lysosomal processing of FLT3-MMAE conjugate (1 µM) in (a) MV4-11 (FLT3-ITD) and (b) THP-1 (FLT3-wt) cells in the presence of bafilomycin A1 (Baf A1) (0–1 nM). Data represented mean ± SD from three independent experiments. Statistical analysis was performed using one-way ANOVA, with significance defined as ***p < 0.001 and ****p < 0.0001.

To determine whether lysosomal activity contributes to the trafficking of FLT3-MMAE conjugate via FLT3-ITD receptor in MV4-11 cells, the cells were pre-treated with varying concentrations of bafilomycin A1 (Baf A1), a selective lysosome inhibitor that disrupts the vacuolar proton pump V-ATPase to neutralise the pH and inhibit lysosomal degradation^36^. After 1 h pre-treatment with Baf A1, the cells were treated with the FLT3-MMAE conjugate, and cytotoxicity was determined using the MTT assay. The results showed no significant difference in the cytotoxicity of the FLT3-MMAE conjugate in MV4-11 cells with or without Baf A1 treatment (Figure 9a). This suggests that inhibiting lysosomal activity did not affect the cellular uptake or lysosomal degradation of the FLT3-MMAE conjugate leading to inefficient MMAE release from the conjugate. In contrast, in THP-1 cells, co-treatment with Baf A1 at 0.1 nM or 1 nM significantly reduced the cytotoxicity of the FLT3–MMAE conjugate (1 µM) compared with treatment with the FLT3–MMAE conjugate alone (Figure 9b), indicating that the conjugate was trafficked to and degraded within lysosomes, resulting in MMAE release in THP-1 cells. These findings were consistent with confocal imaging results showing that FLT3 mAb did not colocalize with lysosomes in FLT3-ITD MV4-11 cells (Figure 5a), indicating that it bypasses the endosomal-lysosomal trafficking pathway. Consequently, the FLT3-MMAE conjugate was not degraded within lysosomes to release the active MMAE payload. Without MMAE release, the drug cannot disrupt microtubule assembly, leading to reduced cell cycle arrest and apoptosis. As a result, the cytotoxic effect of the FLT3-MMAE conjugate in MV4-11 cells was significantly lower than that observed in THP-1 cells (Figure 8), demonstrating that FLT3-ITD mutation exhibited resistance to the FLT3-MMAE conjugate due to receptor accumulation of FLT3-ITD in non-lysosomal compartments.

## Discussion

Antibody-drug conjugates (ADCs) represent a rationally engineered therapeutic strategy that combines the antigen specificity of monoclonal antibodies (mAbs) with the potent cytotoxicity of small-molecule drugs. By selectively delivering cytotoxic payloads to tumour cells, ADCs improve therapeutic selectivity while minimising systemic toxicity. The overall efficacy of ADCs depends not only on high-affinity antibody-antigen binding but also on subsequent intracellular processing, including receptor-mediated internalisation, intracellular trafficking, and lysosomal degradation. Once bound to its target receptor, the ADCs are internalised and trafficked to lysosomes, where the acidic environment and proteolytic enzymes facilitate linker cleavage and payload release^1–4^. Thus, efficient endocytosis and lysosomal processing are essential determinants of ADCs potency and therapeutic index.

In this study, we demonstrated that internalisation of the FLT3 mAb is receptor-dependent and occurs via receptor-mediated endocytosis in both FLT3-wt and FLT3-ITD expressing cells. As shown in Figure 3(c)-f and Figure 4, inhibition of endocytic and energy-dependent processes, including monensin, brefeldin A, and ATP-depleting agents (2-deoxy-D-glucose and sodium azide) inhibited FLT3 mAb internalisation, confirming that antibody uptake into FLT3-expressing cells occurs specifically via FLT3 receptor-mediated endocytosis. Despite similar internalisation efficiency, the intracellular trafficking pathways diverge substantially between these two receptors. In FLT3-ITD MV4-11 cells, the internalised antibody predominantly accumulates in the perinuclear Golgi region, whereas in FLT3-wt THP-1 cells, it localises efficiently to lysosomes (Figure 5). Importantly, this localisation demonstrates that the FLT3 mAb is co-transported with its bound receptor, rather than undergoing independent antibody trafficking, such that the intracellular trafficking pathway of the antibody directly reflects the trafficking behaviour of the FLT3 receptor.

The aberrant Golgi retention observed in FLT3-ITD cells is consistent with a previous report by Yamawaki *et al*. (2021)^37^, which demonstrated that FLT3-ITD predominantly localises to the perinuclear Golgi instead of trafficking to the endosomal-lysosomal pathway. Mechanistically, this mislocalization has been attributed to the constitutive kinase activity of FLT3-ITD, which disrupts normal receptor phosphorylation, post-translational processing, and vesicular transport. These abnormalities impair trafficking to the cell surface and lysosome, causing the receptor and the bound antibody to remain accumulated in the Golgi.

To evaluate the functional consequences of impaired lysosomal trafficking on ADC activity, we designed an FLT3-MMAE conjugate targeting the FLT3 receptor. This ADC consisted of MMAE linked to the FLT3 mAb via a cleavable Val-Cit-PAB linker using *N-*glycan site-specific conjugation. MMAE is a highly potent antimitotic agent^31^^,^^32^. The Val-Cit-PAB linker is widely used in ADC development and is efficiently cleaved by lysosomal cathepsin B, enabling controlled payload release^33^^,^^34^. N-glycan site-specific conjugation enables precise payload attachment at conserved Fc glycosylation sites, which are spatially removed from antigen-binding domains, thereby preserving binding affinity and generating a more homogeneous ADC^38^ compared with conventional random lysine or cysteine conjugation methods^39^.

Consistent with the established ADC mechanism of action, the FLT3-MMAE conjugate exhibited potent, dose-dependent cytotoxicity in FLT3-expressing cells, with minimal activity observed in FLT3-negative K562 cells. Despite comparable antibody binding and internalisation, significantly greater cytotoxicity was observed in FLT3-wt THP-1 cells compared with FLT3-ITD MV4-11 cells (Figure 8). The reduced cytotoxic response in FLT3-ITD cells suggests inefficient payload release, likely due to impaired lysosomal trafficking and insufficient linker cleavage. Although the conjugate is internalised in both cell types, only the FLT3-wt trafficking pathway enables effective lysosomal degradation and MMAE release.

These findings indicate that FLT3-ITD driven aberrant receptor trafficking directly limits the intracellular processing and cytotoxic efficacy of FLT3-targeted ADCs. The impaired lysosomal targeting in FLT3-ITD cells likely compromises the release of MMAE, potentially reducing cytotoxic potency despite effective antibody binding. This mechanism may contribute to the intrinsic resistance of FLT3-ITD-positive leukaemia cells to ADC-based therapies. Overall, these results highlight the importance of receptor trafficking dynamics in ADC design and suggest that therapeutic efficacy may be enhanced by strategies that restore lysosomal routeing of FLT3-ITD or employ linker systems capable of releasing payloads independently of lysosomal degradation.

## Conclusions

In summary, this study demonstrates that the therapeutic efficacy of the FLT3-MMAE conjugate depends on receptor trafficking. Although the FLT3-MMAE conjugate binds and internalises efficiently in both FLT3-wt and FLT3-ITD cells, only the FLT3-wt receptor follows the normal endosomal-lysosomal pathway that enables drug release, while the FLT3-ITD receptor mislocalizes to the Golgi apparatus, preventing lysosomal degradation of the ADC. Consequently, the MMAE payload cannot be efficiently released from the FLT3M-MAE conjugate, leading to reduced cytotoxicity in FLT3-ITD MV4-11 cells. These findings highlight the crucial role of lysosomal targeting in ADC function and suggest that receptor mislocalization can significantly compromise therapeutic potency, resulting in resistance of FLT3-ITD cells to ADC treatment. Accordingly, future FLT3-targeted ADC designs may benefit from linker strategies that enable payload release independently of lysosomal degradation to improve activity in FLT3-ITD AML.

## Supplementary Material

Supplementary data.docx

## Data Availability

The authors confirm that the data supporting the findings of this study are available within the article [and/or] its supplementary materials.
