# Supplementary material for: Mislocalisation of FLT3-ITD receptor contributes to MV4-11 leukaemia cell resistance to antibody-drug conjugate
Source: J Enzyme Inhib Med Chem. 2026 Mar 5;41(1):2638027. doi: 10.1080/14756366.2026.2638027 (PMC12964472; doi:10.1080/14756366.2026.2638027)
Supplement: Supplementary data.docx [file IENZ_A_2638027_SM4033.docx]

**Supplementary Material**

**Mislocalization of FLT3-ITD Receptor Contributes to MV4-11 Leukemic Cell Resistance to Antibody-Drug Conjugate**

**
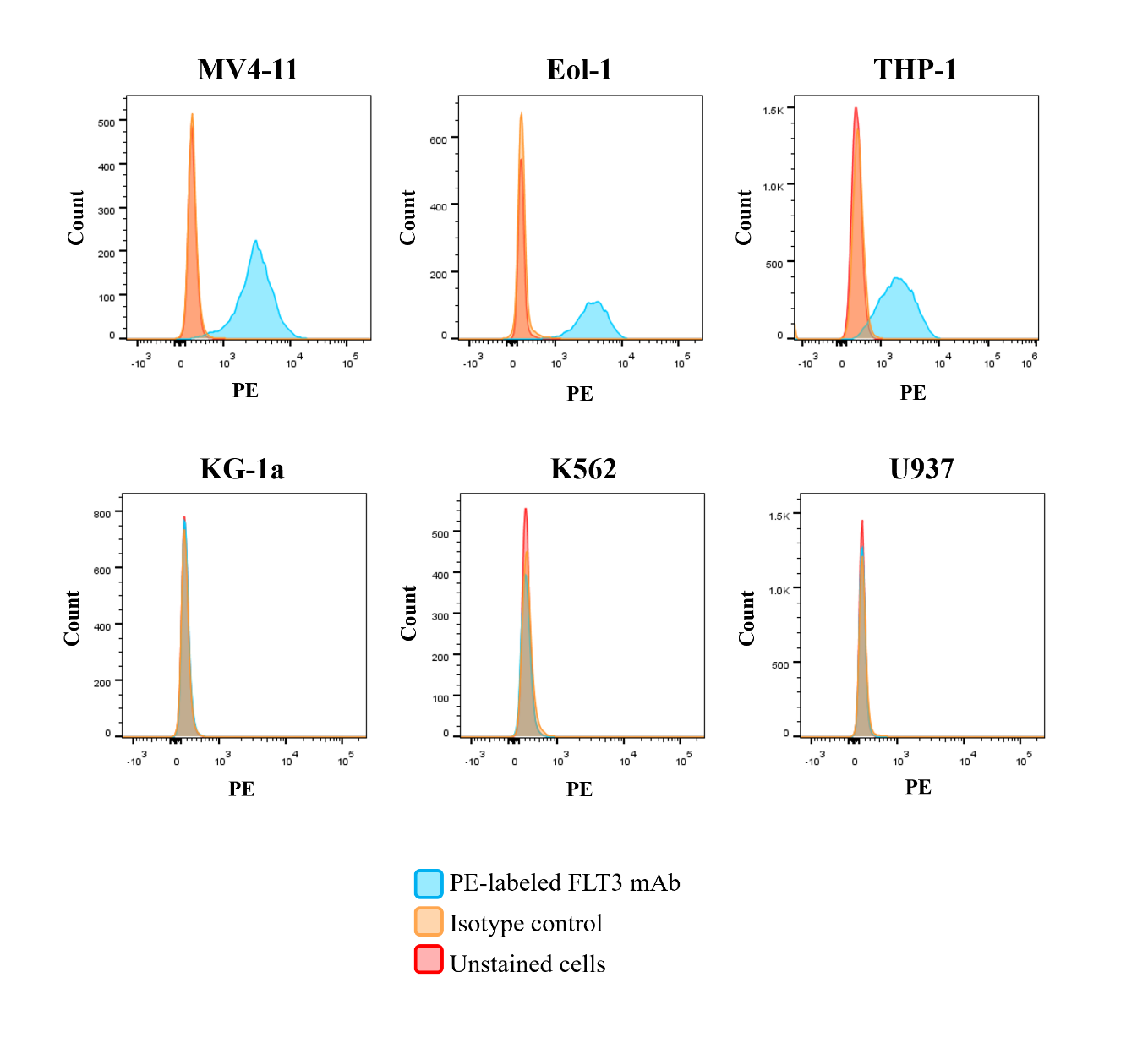
**

**Figure S1.** The FLT3 antibody staining on the cell surface of MV4-11 cells (FLT3-ITD), EO1-1 cells and THP-1 (FLT3-wt), KG-1a cells (leukemic stem-like cells), K562 cells (FLT3-negative), and U937 cells (FLT3-negative). The fluorescent intensities of PE-labelling FLT3 antibody were detected by flow cytometry.
